# Socio-demographic and clinical characteristics of diabetes mellitus in rural Rwanda: time to contextualize the interventions? A cross-sectional study

**DOI:** 10.1186/s12902-020-00660-y

**Published:** 2020-12-10

**Authors:** Charlotte M. Bavuma, Sanctus Musafiri, Pierre-Claver Rutayisire, Loise M. Ng’ang’a, Ruth McQuillan, Sarah H. Wild

**Affiliations:** 1 School of Medicine and Pharmacy, College of Medicine and Health Sciences, University of Rwanda, Kigali, Rwanda; 2 Applied Statistics Department, University of Rwanda, Kigali, Rwanda; 3 Inshuti Mu Buzima, Partners in health-Rwanda, Kigali, Rwanda; 4 Usher Institute, University of Edinburgh, Edinburgh, UK

**Keywords:** Diabetes, Risk factors, Malnutrition, Rural, Rwanda

## Abstract

**Background:**

Existing prevention and treatment strategies target the classic types of diabetes yet this approach might not always be appropriate in some settings where atypical phenotypes exist. This study aims to assess the socio-demographic and clinical characteristics of people with diabetes in rural Rwanda compared to those of urban dwellers.

**Methods:**

A cross-sectional, clinic-based study was conducted in which individuals with diabetes mellitus were consecutively recruited from April 2015 to April 2016. Demographic and clinical data were collected from patient interviews, medical files and physical examinations. Chi-square tests and T-tests were used to compare proportions and means between rural and urban residents.

**Results:**

A total of 472 participants were recruited (mean age 40.2 ± 19.1 years), including 295 women and 315 rural residents. Compared to urban residents, rural residents had lower levels of education, were more likely to be employed in low-income work and to have limited access to running water and electricity. Diabetes was diagnosed at a younger age in rural residents (mean ± SD 32 ± 18 vs 41 ± 17 years; *p* < 0.001). Physical inactivity, family history of diabetes and obesity were significantly less prevalent in rural than in urban individuals (44% vs 66, 14.9% vs 28.7 and 27.6% vs 54.1%, respectively; *p* < 0.001). The frequency of fruit and vegetable consumption was lower in rural than in urban participants. High waist circumference was more prevalent in urban than in rural women and men (75.3% vs 45.5 and 30% vs 6%, respectively; *p* < 0.001). History of childhood under-nutrition was more frequent in rural than in urban individuals (22.5% vs 6.4%; *p* < 0.001).

**Conclusions:**

Characteristics of people with diabetes in rural Rwanda appear to differ from those of individuals with diabetes in urban settings, suggesting that sub-types of diabetes exist in Rwanda. Generic guidelines for diabetes prevention and management may not be appropriate in different populations.

## Background

The prevalence of diabetes mellitus is increasing rapidly worldwide. The number of people with diabetes in the world is expected to rise from 425 million in 2017 to 629 million in 2045, and 79% of these increase is projected to be in low- and middle-income countries (LMICs) [1]. Age-specific prevalence appears to be higher in men than in women in many countries, and the incidence increases with age; type 2 diabetes mellitus is most commonly found in persons over the age of 65 years. Although there is a strong association with obesity in western countries and urban areas in LMICs, diabetes is not uncommon among young and lean people in rural areas in LMICs [2–4]. Furthermore, diabetes onset in Sub-Saharan Africa is reported to occur with more severe symptoms often in under- or normal weight individuals in comparison to Western populations [5]. There is limited information on socio-demographic, etiopathological and clinical profile of diabetes to support development of context specific guidelines for prevention, diagnosis, classification and management of potential atypical sub-types of diabetes mellitus in sub-Saharan Africa and other LMICs [6–8].

The global estimates of the specific prevalence of the main types of diabetes (type 1 diabetes and type 2 diabetes) are limited by the availability of the sophisticated and costly tests that are required to differentiate the sub-types of diabetes, poor awareness of diabetes among the population and health care providers, and limited access to health care facilities especially in rural populations [9, 10]. In LMICs, diabetes is usually classified based on clinical characteristics of individuals diagnosed with diabetes and some individuals may not be easily classified as having a single type of diabetes [11]. Type 2 diabetes mellitus, or insulin-resistant diabetes, is the most common form of diabetes, accounting for more than 90% of the population with diabetes worldwide. As it can be asymptomatic a large proportion is undiagnosed, particularly in low resource settings. The rising prevalence of type 2 diabetes has been attributed to population growth and ageing, urbanization, and increases in obesity and the prevalence of a sedentary lifestyle [2, 12, 13]. Thus, it is thought that a large proportion of type 2 diabetes could be prevented by addressing obesity, physical inactivity and unhealthy dietary habits. However, In African and south Asian population, type 2 diabetes is reported to occur in a significant number of non-overweight individuals [3, 4].

Although the majority of people with a diagnosis of diabetes have type 2 diabetes, up to 25% of individuals with diabetes have been reported to have type 1 diabetes, depending on the population [14–16]. Among the population with type 1 diabetes in Africa, approximately 15% are unclassified or have an atypical phenotype of diabetes, most commonly ketosis-prone atypical diabetes and malnutrition-related diabetes mellitus (MRDM) [17, 18]. In our recent systematic review describing atypical forms of diabetes mellitus in non-European populations in LMICs, we found evidence of MRDM characterized by a type 1 diabetes-like phenotype, a history of childhood malnutrition, underweight at diagnosis, male predominance, young age at diagnosis (third decade), and severe symptoms with high blood glucose without ketosis [6]. Individuals with this diabetes phenotype are typically treated with insulin as a result of their severe hyperglycaemia but do not develop keto-acidosis upon insulin withdrawal. This phenotype might overlap with type 1 diabetes epidemiology in settings in which childhood under-nutrition is prevalent.

Information on diabetes epidemiology and clinical presentations is still limited in rural Africa, where 60–90% of the African population lives. There is likely to be a particularly high proportion of people with undiagnosed diabetes in rural Africa, resulting in an underestimation of the true prevalence of diabetes mellitus in rural areas [19]. Underestimation of diabetes prevalence and limited understanding of demographic and clinical characteristics in the rural population affects prioritization in strategic planning to prevent diabetes and its complications. Current global diabetes prevention and treatment strategies focus on common lifestyle risk factors identified in urban populations such as obesity, alcohol and tobacco consumption and physical inactivity. These strategies may not be effective approaches for diabetes prevention and treatment in rural and poor populations if risk factors for diabetes differ between populations. The majority of guidelines for diabetes care in LMICs are reported not to be appropriate in the local context [20]. The limited existing reports have identified a low prevalence of obesity and high levels of physical activity in rural African populations, suggesting that attempts to prevent diabetes by reducing obesity prevalence and increasing physical activity are likely to be of limited value in this population [21, 22].

In Rwanda, NCDs including diabetes have been recognized as one of health priorities.

In 2013, the overall prevalence of diabetes in Rwanda was 3.2% and the NCDs risk factors step survey showed less prevalent traditional risk factors for diabetes [23]. The current prevalence of diabetes is unknown. Since 2006, the Rwanda Ministry of health started implementing the integrated NCDs clinics at primary care levels to ensure access to health care for all. In these clinics, Diabetes care is held once a week as outpatient care. Individuals diagnosed with diabetes in out- or inpatients wards, are referred in these clinics for follow up. In Rwanda, diabetes is classified as type 1 or type 2 based on clinical characteristics yet some individuals may not fit either type of diabetes. Furthermore, applied guidelines are established based on type 1, type 2 or gestational diabetes. Diabetes care in these NCDs clinics is covered by the community health insurance which is mandatory for each citizen and allows individuals to attend clinic on monthly basis appointment at a minimal cost.

In addition to the effort to improve individual care, some population-based interventions such as “car free day” held twice a month and “Friday physical activity” for public servants are set to raise the awareness of the population about common risk factors for diabetes such as obesity and physical inactivity. Although these measures are necessary and recognized effective to treat and prevent common types of diabetes, atypical presentation of diabetes such as MRDM of which etiopathology and epidemiology are still unknown may not be addressed by such global measures. Characterizing the individuals with diabetes in Rwanda may help to raise the awareness of the decision makers, clinicians and researchers on the needs to contextualize the interventions and to design research to understand the etiopathological mechanism of such atypical diabetes.

One of the global non-communicable disease (NCD) goals adopted by the World Health Assembly in May 2012 is a 25% reduction in premature mortality from NCDs (25 × 25) and a 0% increase in diabetes and obesity by 2025 [24]. The United Nations Sustainable Development Goal 3 is to ensure healthy lives and promote well-being for all people at all ages [16]. If strategies to achieve these goals are to be successful, they must be guided by appropriate, population-specific evidence, including evidence from the majority, impoverished, rural populations in LMICs. The aim of this study is to contribute to this evidence base by describing the frequency of traditional risk factors for diabetes and the socio-demographic and clinical aspects of diabetes in rural compared to urban Rwanda.

## Methods

### Study design

A clinic-based cross-sectional study was conducted from April 2015 to April 2016 in five NCDs clinics of the 39 district hospitals in Rwanda: the Kirehe and Rwinkwavu district hospitals in the Eastern Province, the Butaro and Musanze district hospitals in the Northern Province and the Kabgayi district hospital in the Southern Province. These five health facilities were purposively selected because they have separate diabetes clinics and well-defined diabetic clinic days (once a week) and operate a standardized medical recording system, facilitating the logistics of data collection. Two of the hospitals (Rwinkwavu and Butaro) are located in remote rural areas, while the other three are located in urban areas but serve a mix of rural and urban populations.

We consecutively recruited all people with diabetes (newly diagnosed and prevalent cases) attending the NCDs clinics in the above district hospitals for their routine diabetes clinic appointments during the period of study. We made sure one individual is enrolled once by recording their clinic identity number as individuals with diabetes are given a monthly appointment.

### Inclusion and exclusion criteria

We included women and men of all ages with all types of diabetes who consented to participate. Women who developed diabetes during pregnancy were excluded from the study as well as people with diabetes with known causes such as pancreatic cancer, pancreatitis or endocrine diseases.

### Data collection

Demographic, socio-economic and clinical data from patient interviews, physical measurements and medical records reviews were collected using a paper case report form (CRF). Questions and physical measurement techniques from the WHO (World Health Organization) step-wise NCD risk factors survey were used to determine modifiable risk factors [25]. Four experienced nurses and one medical students were each assigned to each site for weekly data collection on the diabetes day clinic under supervision of the principal investigator to ensure the completeness of the CRF.

### Definition and variables measurement

Socio-demographic variables included the reported age (at diagnosis and enrolment time), sex, reported residence (rural and urban as defined by the National Institute of Statistics of Rwanda). Participants were interviewed regarding access to electricity and running water, type of work occupation categorized in low and high income activities (based on their monthly income) and medical insurance coverage .

Key risk factors for diabetes included: physical activity intensity categorized in vigorous physical activity (activities that cause a large increase in breathing or heart rate; e.g.: digging), moderate physical activity (activities that cause a small increase in breathing or heart rate, example: carrying light loads for at least 10 min continuously) and low physical activity, e.g.: physical inactivity), reported smoking status, alcohol consumption, vegetable and fruits consumption.

Physical measurements: systolic and diastolic blood pressure were measured at all sites using an automated blood pressure after 15 min rest and the average of three readings was recorded and considered for the analysis. The weight was measured in light clothes using an electronic patient weighing machine with a laser for the height measurement. The Body Mass Index (BMI) was the result of weight in kilograms divided by the square of height in meters (Kg/m^2^). The waist circumference was measured using a tape placed around the middle of the abdomen (above hipbones) in a standing position and was expressed in centimetres (cm).

Clinical variables included: The recorded or reported history of childhood undernutrition, reported family history of diabetes, symptoms and blood glucose when received the diagnosis of diabetes, initial and ongoing treatment when enrolled for the study.

Venous blood samples were collected for the glycated hemoglobin and the blood glucose at the time of enrolment to the study.

### Statistical analysis

Patients were characterized as either rural or urban residents based on the location of their reported domicile. Chi-squared tests were used to compare frequencies and proportions of categorical variables. Mean values of continuous variables of rural and urban participants were compared using the t-test. A significance level of 5% was set for all tests. Data entry and analysis were performed using SPSS (statistical package for the Social Sciences) software version 21.

## Results

### Socio-demographic characteristics of participants

A total of 472 participants with diabetes fulfilling the inclusion criteria, were recruited (62, 177, 65, 94 and 74 respectively from Butaro, Kabgayi, Kirehe, Ruhengeri and Rwinkwavu) and enrolled in the study, with a response rate of 100% in both sex, of which 62.5% were women. Two women who had diabetes diagnosis in the third trimester of pregnancy and one men with acromegaly were excluded from the study. The majority of participants (66.7%) were rural dwellers. The mean ± standard deviation (SD) age of the participants was 40.2 ± 19.1 years, with an age range of 5 to 86 years. Rural participants had a significantly lower mean ± SD age than urban residents (37 ± 19 vs 47 ± 18, respectively; *p* < 0.001). The duration of diabetes ranged from less than 1 year to 22 years, and the mean ± SD duration was 2.7 ± 2.5 years.

The sex distribution was similar in both the urban and rural populations, with a female predominance (Table 1). A highest proportion of rural participants was in their second or third decade while in urban residents, majority was in the fifth-sixth decade (Table 1). Rural residents had significantly lower levels of education and were significantly more likely to be in low-income employment than urban residents; however, the majority of both groups were in the low-income and low-education level categories, and differences between urban and rural dwellers were small (see Table 1). As shown in Table 1, compared to urban residents, rural individuals had limited access to running water and electricity. and higher proportions of rural residents reported using herbal medicine for diabetes-related symptoms before the diagnosis of diabetes at a modern hospital despite a high uniformly medical insurance coverage, which could facilitate accessibility to modern medical care in both settings (see Table 1).

**Table 1:** Socio-demographic profile of survey participants with diabetes from five district hospitals in Rwanda, 2015–2016

Variable	Rural dwellers		Urban dwellers		***P*** value
	N	%	N	%	
**Sex**					
Men	107	36.9	70	38.5	0.403
Women	183	63.1	112	62.5	
**Age range (years)**					
< 15	7	2.4	1	0.5	< 0.001
15–29	145	50.0	49	26.9	
30–44	35	12.1	26	14.3	
45–59	53	18.3	61	33.5	
60–74	45	15.5	36	19.8	
≥ 75	5	1.7	9	4.9	
**Education level**^a^					
Low	269	92.8	156	85.7	0.011
High	21	7.2	26	14.3	
**Herbal medicine use**	36	12.4	9	4.9	0.004
**Access to electricity**	67	23.1	164	90.1	< 0.001
**Access to running water**	124	42.8	168	92.3	< 0.001
**Work type**^b^					
Low-income	261	90.0	150	82.4	0.030
High-income	14	4.8	20	11.0	
Missing data	15	5.2	12	6.6	
**Medical insurance coverage**	290	100	181	99.5	0.386

^a^Low education level includes illiterate to incomplete secondary school categories; high education level includes those who completed secondary school or higher

^b^Low-income work includes unemployment, subsistence farming, non-paid volunteers and students; high-income work includes non-government organizations (NGOs) employees, governmental institution employees and all activities generating more than 100,000 Rwandan francs (approximately 100 USD) per month

### Traditional risk factors for diabetes

Rural residents received their diagnoses of diabetes at a younger mean age than urban residents; the mean ± SD age was 32 ± 18 for rural residents vs 41 ± 17 years for urban residents. A family history of diabetes, obesity and high waist circumference were significantly less common in rural residents than in urban residents with diabetes (see Table 2). Rural dwellers appeared to be significantly more physically active than urban dwellers (Table 2). The proportion of ever smokers was significantly higher in urban residents than in rural participants. Rural dwellers reported less frequent fruit and vegetable consumption than urban participants (the mean weekly number of fruits and vegetables consumed was 1.5 ± 1.7 vs 2.8 ± 2.5; *p* < 0.001 and 4.5 ± 2.4 vs 5.4 ± 2.2; *p* < 0.001, respectively). The mean systolic and diastolic blood pressures were lower in rural participants than in urban individuals (127 ± 20 vs 136 ± 21 mmHg, respectively; *p* < 0.001).

**Table 2:** Distribution of traditional risk factors among survey participants with diabetes attending five district hospitals in Rwanda in 2015–2016 by rural/urban residence status

Variables	Rural		Urban		*P* value
	N	%	N	%	
Physical activity intensity*					
Vigorous	126	40.0	28	17.8	< 0.001
Moderate	49	15.6	25	15.9	
Low	140	44.4	104	66.3	
Reported family history of diabetes					
Positive	47	14.9	45	28.7	0.001
BMI (kg/m2)					
< 18.5	50	15.9	5	3.2	< 0.001
19–24.9	178	56.5	67	42.7	
25–29.9	62	19.7	54	34.4	
≥ 30	25	7.9	31	19.7	
Systolic blood pressure (mmHg)					
< 120	118	37.8	34	21.7	0.001
120–139	122	39.1	65	41.4	
140–159	45	14.4	34	21.7	
≥ 160	27	8.7	24	15.3	
Diastolic blood pressure					
< 80	197	63.1	69	43.9	0.001
80–89	67	21.5	50	31.8	
90–99	33	10.6	25	15.9	
≥ 100	15	4.8	13	8.3	
Tobacco use					
Never smoked	249	79.0	105	66.9	0.01
Ever smoked	66	21.0	52	33.1	
Alcohol consumption					
Never drank alcohol	156	49.5	61	38.9	0.087
Stopped over 12 months ago	104	33.0	64	40.8	
Stopped less than 12 months ago	23	7.3	9	5.7	
Current alcohol consumer	32	10.2	23	14.6	
Waist circumference					
Women (> 80 cm)	90	45.5	73	75.3	< 0.001
Men (> 94 cm)	7	6.0	18	30.0	< 0.001

*N* number of participants ^*^Vigorous physical activity: activities that cause a large increase in breathing or heart rate (example, digging), moderate physical activity: activities that cause a small increase in breathing or heart rate (example, carrying light loads) for at least 10 min continuously [25], low intensity activity: physical inactivity

### Clinical characteristics of the participants

Unusual high proportion of participants having type 1 diabetes is observed in overall participants (47.4% of participants classified as type 1 diabetes, 44.5% as type 2 diabetes and 8.1% of participants were unclassified). The proportion of type 1 diabetes and the frequency of childhood malnutrition were higher in rural residents than in urban individuals (see Table 3). Diabetes duration was shorter in rural residents than in urban participants (mean duration was 56.0 ± 52 months in rural participants vs 83.0 ± 71 months in urban individuals). Most rural individuals required insulin at diagnosis and at study enrolment (Table 3). Rural dwellers were diagnosed with higher blood glucose than those in urban settings (mean fasting blood glucose was 476 ± 148 mg/dl (26.4 ± 8.2 mmol/l) in rural residents vs 386.0 ± 149.4 mg/dl (21.4 ± 8.3 mmol/l) in urban participants; *p* < 0.001, respectively). Severe symptoms, such as unconsciousness at diagnosis, were more reported by rural participants than urban participants. (Table 3). The mean HbA1c at study enrolment was higher in rural than in urban individuals (8.9 ± 2.7% vs 8.2 ± 2.3%, respectively; CI: 0.7 (0.2–1.2)).

**Table 3:** Distribution of clinical characteristics among survey participants with diabetes attending five district hospitals in Rwanda from 2015 to 2016 by rural/urban residence

Variables	Rural		Urban		D (95% CI)	***P*** value
	N	%	N	%		
**Type of diabetes**						
Type 1	185	58.7	39	24.8	33.9 (24.7–41.9)	< 0.001
Type 2	104	33.0	106	67.5	34.5 (25.1–42.9)	< 0.001
Unclassified	26	8.3	12	7.6	0.7 (5.1–5.5)	0.792
**Reported history of childhood malnutrition**	71	22.5	10	6.4	16.1 (9.5–21.8)	< 0.001
**Age range**						
≤ 30 years	171	54.3	37	23.6	30.7 (21.6–38.7)	< 0.001
> 30 years	144	45.7	120	76.4		
Diabetes duration						
< 12 months	32	10.2	15	9.6	0.6 (−5.7–5.9)	0.838
12–60 months	176	55.9	62	39.4	16.5 (6.9–25.5)	< 0.001
> 60 months	107	34.0	80	51.0	17 (7.5–26.2)	< 0.001
**Blood glucose at diagnosis**						
< 250 mg/dl	23	7.3	30	19.1	11.8 (5.4–19.0)	< 0.001
250–400 mg/dl	69	21.9	50	31.8	9.9 (1.5–18.6)	0.019
> 400 mg/dl	180	57.1	60	38.2	18.9 (9.3–27.8)	< 0.001
Unknown	43	13.7	17	10.8	2.9 (−3.8–8.6)	0.373
**Blood glucose of participant at the time of study recruitment**						
< 250 mg/dl	248	78.7	139	88.5	9.8 (2.5–16.1)	0.009
250–400 mg/dl	47	14.9	13	8.3	6.6 (0.2–12.1)	0.043
> 400 mg/dl	20	6.4	5	3.2	3.2 (−1.4–6.9)	0.145
**Number of participants with Comatose state at diagnosis**	82	26.0	16	10.2	15.8 (8.5–22.2)	< 0.001
**Number of individuals requiring Insulin at diagnosis**	214	67.9	63	40.1	27.9 (18.4–36.7)	< 0.001
**Number of individuals on Insulin treatment at the time of participants recruitment**	203	64.4	54	34.4	30 (20.5–38.6)	< 0.001

*N* number of participants

*D* difference

## Discussion

The sex distribution was similar between rural and urban individuals, with a female predominance. Although our study was not a prevalence study, differences in diabetes prevalence by sex have been reported to be variable depending on the population and setting [26]. In some African populations, diabetes prevalence is higher in men [27], and in others diabetes prevalence is higher in women [28]. In Cameroon, a similar sex distribution in diabetes prevalence has been reported in rural populations, while in urban individuals, a female predominance was noticed [29]. This variability in the sex-specific prevalence of diabetes might be related to differences in exposure to the risk factors for diabetes by sex; for example, the Rwanda NCD risk factors survey revealed that obesity and overweight are more prevalent in women than in men [30]. This variability implies the need for specific population-based assessments of sex differences in terms of diabetes burden for targeted and need-based interventions to address the diabetes burden.

We found that diabetes was diagnosed at a relatively young age in our study population, which was most noticeable in rural individuals. Furthermore, rural residents were younger at the time of study enrolment. The age distribution among rural residents was consistent with patterns reported in other African populations [1, 31] and in a south Asian population [32], and the age distribution was inconsistent with the findings in Western and urban African populations, in which larger proportions of older people were found among people with diabetes [31, 33]. The age distribution among rural and LMIC populations with diabetes in general might be explained by higher proportions of misclassified type 1 diabetes or so-called “malnutrition-related diabetes”, or other atypical diabetes subtypes in underserved settings for which the onset has been reported to be in the second and the third decade of life [34–36]. However, the lower proportion of diabetes in rural older age individuals could also be explained by the early diabetes related mortality leading to poorer survival rate in more disadvantaged populations [37, 38] and short life expectancy in individuals with diabetes living in poverty [39].

We found that rural and urban dwellers were uniformly and highly covered by the medical insurance at a rate greater than 95%. This is similar to the general population since the medical coverage has been made mandatory for all Rwandan citizens in 2008. Most rural residents reported being in low-income work, having limited access to running water and electricity, more common use of herbal medicine for high blood glucose symptoms and less fruit and vegetable consumption when compared to urban residents. We were not able to describe the association of socio-economic condition with diabetes prevalence because population data were not available. At later stages of the epidemiologic transition, low socio-economic status is associated with an increased risk of NCDs such as diabetes mellitus, cancers and cardiovascular diseases [40]. Poverty and food insecurity might contribute to the increasing prevalence of diabetes in some rural African settings in which diabetes prevalence is reported to exceed the diabetes prevalence in urban areas [31]. In addition to the fact that poverty might contribute to the onset of diabetes potentially through foetal and childhood under-nutrition or obesity in later life, poverty is reported to be a factor related to unequal access to care [41]. More importantly, even though there was no difference in health insurance coverage and if there were dedicated diabetes clinics in rural hospitals in Rwanda, poverty would make it more difficult for people with diabetes to keep themselves healthy. This is because of limited access to a healthy diet, electricity, a refrigerator to store insulin, and running water to keep injection sites clean as well as the ability to travel for specialist care, such as eye care. The impact of poverty and its consequences for the burden of diabetes and its complications should be explored further in low-income countries. In addition to poverty related healthy diet inaccessibility, disparities observed in fruit and vegetables consumption can be explained by limited knowledge, awareness and practice towards healthy diet. Despite Rwanda effort to promote healthy diet, the rural population practice may differ from the knowledge due to social and culture dimension of food observed in other similar countries [42].

We found that traditional risk factors for type 2 diabetes, such as family history of diabetes, obesity and physical inactivity, were less prevalent among rural individuals. Central obesity was prevalent in both groups but was less common in rural residents. This result is consistent with the findings of other reports from LMICs in which the increasing prevalence of diabetes did not match the low prevalence of common risk factors for diabetes [32, 43, 44]. This finding suggests that there might be other factors contributing to the increase in diabetes prevalence in low-income settings.

We found a higher prevalence of reported childhood under-nutrition among rural than among urban residents. It has long been suggested that chronic under-nutrition is associated with impaired insulin secretion [45]. To our knowledge, childhood under-nutrition as a risk factor for diabetes in adulthood has been given limited attention in Sub-Saharan Africa, where its prevalence is reported to be high, especially in East Africa [46], where evidence suggests that the prevalence of diabetes in the poorest population exceeds the prevalence in less poor populations [31]. In urban settings, traditional risk factors remain the main drivers of the rapid increase in diabetes [27, 28].

We observed an unusually high prevalence of type 1 diabetes among our study participants, particularly among rural dwellers. Furthermore, most participants, particularly those from rural areas, reported insulin requirements from diagnosis. Our finding corroborate other studies’ results in LMIC where Type 1 and type 2 diabetes have been reported to be equally common [47] in contrast with Western countries where type 2 diabetes is considerably more common. The over-representation of type 1 diabetes could reflect the limitation of clinic-based nature of the study; people with type 2 diabetes might have participated in fewer clinic visits or received their care in other settings. In our case, people with diabetes are given appointments to attend the NCD clinics on a monthly basis for prescription renewal and follow up, with active retrieval of those who were lost to follow-up, regardless of the type of diabetes. Furthermore, we recruited participants in various health facilities on different diabetes clinic days over a whole year to overcome potential selection bias. Diabetes classification is usually based on clinical presentations in our clinics, and atypical diabetes with type 1-like phenotypes such as MRDM and ketosis-prone type 2 diabetes could have been misclassified as type 1 diabetes. This misclassification may have negative impact on the necessity to understand the aetio-pathology of the above atypical phenotypes and on the decision making for treatment and prevention. Furthermore, lean, young individuals with a history of childhood under nutrition and without classic risk factors for type 2 diabetes or classical features of type 1 diabetes do not fit any type of the diabetes classes mentioned in 1999 World Health Organization (WHO) diabetes classification although could be assigned to the unclassified group in the 2019 updated classification [48].

Although our study population was uniformly well covered by medical insurance, more rural individuals than urban participants reported severe hyperglycaemia at diagnosis and use of herbal medicine, and their diabetes was less well controlled. Limited access to diabetes care, easy accessibility to traditional healers, lack of resources and frequent lack of stock of modern diabetes drugs have been reported to be the reasons for herbal medicine use and poor quality of diabetes care in LMICs [49, 50]. Furthermore, the difference in timely healthcare seeking behavior in rural vs urban areas might be explained by less awareness and knowledge about the nature of diabetes and treatment measures in rural settings given that the cost and geographical access limitations reported in other limited resources countries are mitigated by a higher medical coverage and the nature of Rwanda health system promoting near home health care. There is a need to identify other barriers to quality diabetes care in the setting in which universal medical coverage is maximized and diabetes care decentralization to lower levels of the health system is established to improve equitable access to care.

### Study limitations

This study raises an important challenge faced by clinicians who are dealing with diabetes classification, treatment and prevention in rural Rwanda. In addition it might stimulate the research need to understand the risk factors for diabetes and the re-consideration of MRDM in Rwanda. However it has some limitations, firstly the nature of the study design being a clinic- based study may cause a selection bias but we made it multi-centers including clinics in various provinces of Rwanda.

## Conclusion

The findings of this study shows that individuals with diabetes in rural Rwanda compared to urban settings, have particular socio-demographic and clinical profile characterized by low socio-economic conditions, young age of onset, leanness, lower prevalence of traditional risk factors for type 2 diabetes, higher prevalence of reported childhood under-nutrition and an unusually high prevalence of type 1 diabetes which reflect the challenges faced by healthcare providers in diabetes diagnosis, classification and clinical care decision making in Rwanda. Under-nutrition and over-nutrition as well as poverty might play an important role in the burden of diabetes in Rwanda. Further studies are required to assess risk factors for sub-types of diabetes and their aetio-pathology in rural Rwanda and other low-income settings and to identify effective interventions to inform guidelines to prevent and treat all forms of diabetes in LMICs. There is a particular need to establish assess the burden caused by MRDM and other atypical types of diabetes and appropriate approaches to their primary and secondary prevention. Rwanda should re-visit the diabetes classification to include the atypical phenotypes of diabetes and to consider these phenotypes in the diabetes registry for surveillance and monitoring.

This study shows rural vs urban discrepancy in timely healthcare seeking and healthy diet practice. There is a need to assess the barriers to the adequate healthy diet and timely medical check-up practice in rural communities to inform population based health education to address diet behaviour and access to care.

## Data Availability

The datasets analysed during this study are available from corresponding author on reasonable request.

## Abbreviations

LMICs: Low- and middle income countries
MRDM: Malnutrition-related diabetes
NCDs: Non-communicable diseases
e.g: Exempli grati (For example)
CRF: case report form
WHO: World Health Organization
HbA1c: Glycated haemoglobin
SPSS: Statistical package for the social sciences
SD: Standard deviation
vs: versus
BMI: Body Mass Index
WC: Waist circumference
